# Precise Geoid Determination in the Eastern Swiss Alps Using Geodetic Astronomy and GNSS/Leveling Methods

**DOI:** 10.3390/s24217072

**Published:** 2024-11-02

**Authors:** Müge Albayrak, Urs Marti, Daniel Willi, Sébastien Guillaume, Ryan A. Hardy

**Affiliations:** 1 School of Civil and Construction Engineering, Oregon State University, Corvallis, OR 97331, USA; 2 School of Management and Engineering Vaud (HEIG-VD), University of Applied Sciences and Arts Western Switzerland (HES-SO), 1400 Yverdon-les-Bains, Switzerland; sebastien.guillaume@heig-vd.ch; 3 Federal Office of Topography Swisstopo, 3084 Bern, Switzerland; urs.marti@swisstopo.ch (U.M.); daniel.willi@swisstopo.ch (D.W.); 4 NOAA National Geodetic Survey, Silver Spring, MD 20910, USA; ryan.hardy@noaa.gov

**Keywords:** deflections of the vertical, geodetic astronomy, GNSS/leveling, geoid, CHGeo2004

## Abstract

Astrogeodetic deflections of the vertical (DoVs) are close indicators of the slope of the geoid. Thus, DoVs observed along horizontal profiles may be integrated to create geoid undulation profiles. In this study, we collected DoV data in the Eastern Swiss Alps using a Swiss Digital Zenith Camera, the COmpact DIgital Astrometric Camera (CODIAC), and two total station-based QDaedalus systems. In the mountainous terrain of the Eastern Swiss Alps, the geoid profile was established at 15 benchmarks over a two-week period in June 2021. The elevation along the profile ranges from 1185 to 1800 m, with benchmark spacing ranging from 0.55 km to 2.10 km. The DoV, gravity, GNSS, and levelling measurements were conducted on these 15 benchmarks. The collected gravity data were primarily used for corrections of the DoV-based geoid profiles, accounting for variations in station height and the geoid-quasigeoid separation. The GNSS/levelling and DoV data were both used to compute geoid heights. These geoid heights are compared with the Swiss Geoid Model 2004 (CHGeo2004) and two global gravity field models (EGM2008 and XGM2019e). Our study demonstrates that absolute geoid heights derived from GNSS/leveling data achieve centimeter-level accuracy, underscoring the precision of this method. Comparisons with CHGeo2004 predictions reveal a strong correlation, closely aligning with both GNSS/leveling and DoV-derived results. Additionally, the differential geoid height analysis highlights localized variations in the geoid surface, further validating the robustness of CHGeo2004 in capturing fine-scale geoid heights. These findings confirm the reliability of both absolute and differential geoid height calculations for precise geoid modeling in complex mountainous terrains.

## 1. Introduction

The geoid is Earth’s equipotential surface that best fits the mean sea level. It models how fluids (e.g., water, ocean, ice sheets) flow and, in turn, are shaped by mass changes; it also may be used to define a vertical datum (e.g., [1]). Therefore, the geoid forms the basis of height systems. To obtain the physical heights of a benchmark practically, Global Navigation Satellite Systems (GNSS)-derived ellipsoidal heights (h) must be converted into orthometric heights (H) using an appropriate geoid model (e.g., [2,3]). Orthometric height may be converted into a geopotential value, normal height, or dynamic height depending on the needs of the user. A geoid model can be optimally determined by combining different geodetic observation types to obtain the “best” geoid solution, leveraging the disparate datasets, by solving the geodetic boundary value problem [4]. In this paper, we describe a 2021 project to collect geodetic (GNSS and leveling), gravimetric (gravity), and astrogeodetic (deflections of the vertical (DoV)) data in the field to determine the geoid profile in the challenging mountainous Surses region (Canton de Grisons) in the Eastern Swiss Alps.

Geopotential differences may be obtained from the path integral of the gravity vector through space. Astrogeodetic DoVs determine the direction of the gravity vector [5,6], while relative gravity measurements determine its magnitude. DoVs are close indicators of the slope of the geoid. If DoV observations are carried out along horizontal lines, geoid undulation profiles, which are prerequisites for height system modernization, may be obtained via direct integration [7]. DoVs are the most difficult data to observe in comparison to other datasets (GNSS, leveling, gravity, etc.). The advantage of DoV data is that they are independent from all other datasets, and therefore do not introduce any biases in comparative analysis. Thus, they are capable of validating geoid models from other datasets, revealing possible shortcomings in official geoid and regional gravity field models and height systems, and delivering important accuracy estimates for those official models [8]. The best examples of recent geoid model validation using DoV data are the three successful campaigns of the “Geoid Slope Validation Surveys (GSVSs)” in the US [8,9,10]. As Jekeli [7] describes, DoV data are generally excluded from modern global gravity field model (e.g., Earth Gravitational Model (EGM)) determinations; therefore, prior DoV observations show that DoV data are a promising tool to support validating existing global gravity field models [11]. DoV data have been used together with gravity, GNSS, and leveling data to determine hybrid geoids in Switzerland (Swiss Geoid Model 2004 (CHGeo2004)) [12], Austria [13], and Hungary [14]. To the best of our knowledge, these three geoids are the only highly precise hybrid geoid models that have been combined with DoV data.

Nowhere have astrogeodetic methods been used more intensively than in Switzerland. Astrogeodetic observations are the most suitable method for geoid determination in mountainous regions such as the Swiss Alps where satellite-based geoid model accuracy is lower in mountainous areas. Astrogeodetic methods can determine the geoid using profile measurements alone, which is compatible with survey designs confined to mountain valleys. DoVs are also relatively insensitive to height changes, which is a source of error in rugged terrain. By contrast, gravimeter measurements require sampling in two dimensions to determine the geoid and they are extremely sensitive to errors in instrument height. GNSS/leveling is another method for determination of the geoid, but it is limited by the labor-intensiveness of leveling, the accuracy of ellipsoidal heights, and the lengthy GNSS observations occupation times required.

For the continuity of Swiss astrogeodetic observations, a Swiss digital zenith camera, the DIgital Astronomical DEflection Measuring (DIADEM), developed at ETH Zurich, has been updated as the COmpact DIgital Astronometric Camera (CODIAC) in parallel with the development of more advanced components [15]. Additionally, the first robotic total station-based astrogeodetic system, QDaedalus, was also developed at ETH Zurich [16,17]. To collect the DoV data in the Eastern Swiss Alps, we used the CODIAC and two Leica TS60 robotic total station-based QDaedalus systems. The test measurements of these three systems were done before and after the observation campaigns [18,19,20]. We benefited from utilizing two types of astrogeodetic instruments, as the QDaedalus system allowed us to carry out observations in terrain inaccessible by the CODIAC. More information about the CODIAC and QDaedalus systems and the Surses region DoV observations can be found in [19].

For this Swiss Alps study, the astronomical geoid profile was established along an N-S astrogeodetic line spanning approximately 18 km. Since astronomical geoid profiles achieve higher accuracy over shorter distances, we aimed for a smaller station spacing of approximately 1 km. Many previous astrogeodetic profiles have station spacing ranging from a minimum of 50 m but not exceeding 1 km [21,22,23,24]. However, maintaining an exact 1 km spacing is not always feasible in mountainous regions.

As with the DoV observations, the GNSS, leveling, and gravimeter measurements were conducted using conventional instruments specifically developed for these kinds of observations (see Section 2). The gravimeter measurements were used to inform elevation-dependent corrections to the astrogeodetic geoid profile. The gravimeter measurements give the magnitude of the gravity vector at each station, whose directional components were established astrogeodetically. The collected gravity data were primarily used to correct the DoV data. Since the benchmarks in the profile were located around artificial reservoirs, Lake Marmorera, and both the DoV and gravity data were affected by temporal variations in water levels. The other data were used to compute geoid heights. Therefore, two different geoid height computations were performed: (i) using geodetic data from GNSS and leveling measurements, and (ii) using DoV observations. Consequently, two conventional methodologies were carried out to determine geoid heights from the collect-ed data: GNSS/leveling and astronomical leveling (based on DoV) methods. We note that astronomical leveling measures geoid height changes by integrating measurements of geopotential gradients, while the starting point for GNSS/leveling is finely integrated geo-potential differences and absolute ellipsoidal heights. As the astronomical technique is a fundamentally differential measurement, we examined our geoid profiles both in terms of absolute geoid heights and differential geoid increments. These results were also com-pared with CHGeo2004 and two global gravity field models, the Earth Gravitational Mod-el 2008 (EGM2008) [25] and the combined global gravity field model XGM2019e [26].

## 2. Measurements and Accuracy of the Individually Collected Field Data

**Figure 1:**
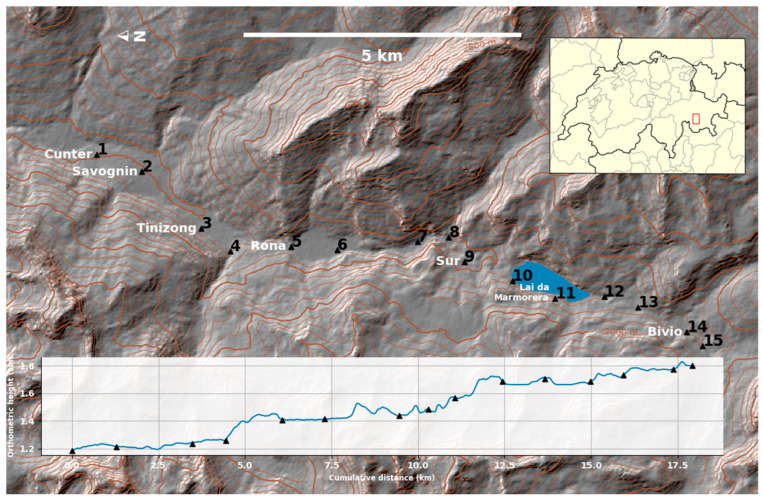
Surses region geoid profile in the Eastern Swiss Alps, where DoV, GNSS, leveling, and gravity observations were carried out on 15 benchmarks. The Shuttle Radar Topography Mission (SRTM) is visualized along the profile.

In the Eastern Swiss Alps, the geoid profile was established in 15 benchmarks from Cunter to Plan Buel along the Julier Valley (Surses region) during a two-week period in June 2021 (Figure 1). DoV, GNSS, leveling, and gravity observations were carried out on these benchmarks with an elevation ranging between 1185 to 1800 m and a benchmark spacing ranging from 0.55 km to 2.10 km (1.28 km, on average). The benchmarks were chosen mainly for astrogeodetic and GNSS measurement suitability since the sky visibility requirements of these extraterrestrial observations are more restrictive than the requirements for leveling and gravity. The criteria for benchmark selection were: (1) tripod installation capability at the benchmark (the benchmark should not be on a wall or on a rock face); (2) GNSS measurement suitability (not too close to buildings, trees, or high voltage power lines); and, (3) ability to perform visual observations of zenith angles up to 30° and Polaris line of sight without obstruction (relevant for the QDaedalus system). All observations were conducted separately using the conventional procedure: the GNSS, leveling, and DoV observations were conducted on the benchmarks, while the gravity observations were conducted beside the benchmarks. The GNSS and DoV observation procedures for the Surses region campaign were published alongside the DoV results in [19].

### 2.1. Astrogeodetic Deflections of the Vertical (DoV) Observations

To obtain the astrogeodetic DoV components, astronomical and geodetic coordinates must be obtained at the same benchmarks. Astronomical latitudes (Φ) and longitudes (Λ) were observed with astronomical observations using one CODIAC system and two Leica robotic TS60 total station-based QDaedalus systems, while the geodetic latitudes (φ) and longitudes (λ) were obtained through double-frequency GNSS measurements (see Section 2.2). As a result, the N-S (ξ) and E-W (η) DoV components were calculated as follows (e.g., [27,28]):

(1) ξ=Φ−φ

(2) η=(Λ−λ)cos⁡φ

Total DoVs can be calculated as:

(3) $$\varepsilon =\sqrt{\xi^{2}+\eta^{2}}$$

The QDaedalus systems and CODIAC were deployed on thirteen (over five nights) and two (one night) benchmarks, respectively. The DoV data for each benchmark were obtained for the specific system used at the given benchmark. The QDaedalus observations were executed between two and four series (weather dependent) of about 15 min (we define a “series” as a discrete observation sequence) per occupation session, while the CODIAC observations were conducted in two sessions, each session consisting of four series of observations (about 10 min per session).

The QDaedalus and CODIAC post-processing can be performed 24 h after the observations are completed by using the more precise Earth Orientation Parameters (EOPs), but we preferred to conduct a second post-processing after a month to utilize the final EOPs, which are crucial to calculate more precise DoV data for CODIAC. Having precise GNSS coordinates is also significant for CODIAC (see Section 2.2). As a result of the adjustment, the QDaedalus DoV data individual session standard deviations (SDs) at the 13 benchmarks are 0.04″–0.22″ for the N-S and 0.01″–0.20″ for the E-W DoV components, while CODIAC DoV data individual session SDs at the two benchmarks are 0.02″ for both DoV components [19]. The DoV data and their respective SDs can be found in Appendix A.

### 2.2. GNSS Measurement

The GNSS measurements were conducted using two different receivers: four Trimble R8 Model 3 receivers with integrated antennas (Trimble GmbH, Raunheim, Germany) and two JAVAD SIGMA-G3T receivers with JAVAD GrAnt-G3T antennas (JAVAD GNSS, San Jose, CA, USA). Static measurements were carried out at each station in two separate sessions of 5–16 h.

The post-processing of each benchmark was performed using Bernese GNSS software v5.2 [29]. This software was selected for its high precision in geodetic applications, particularly for tasks involving rigorous GNSS data analysis. A relative positioning solution was performed using the “SAM2” station in Samedan from the Automated GNSS Network of Switzerland (AGNES), with baseline lengths of 18–24 km. Rapid products (orbits, clocks, earth rotation parameters) and differential code biases from the Center for Orbit Determination in Europe (CODE) were utilized [30]. Modelling of the troposphere was performed by the Vienna Mapping Function 1 (VMF1). The global ionospheric maps provided by the CODE are used for ionospheric corrections. The ocean loading displacements are retrieved from the Chalmers University ocean loading provider. GPS-only data were considered for the final processing.

The estimated GNSS coordinates’ root mean square (RMS) results per day were 15–20 mm for the East, North, and Up components [19,31]. The geodetic coordinates in the ITRF2014 reference frame at the reference epoch of 2021.67 can be found in Appendix A. These coordinates were used for the calculation of the DoV data and geoid height from GNSS/leveling and astronomical leveling.

### 2.3. Geometric Leveling Measurement

The geometric leveling measurements were only conducted locally between the GNSS station and the nearest leveling benchmark with the digital level *Leica DNA03*. The leveling measurements were conducted as double leveling measurements performed with the setting “Back (B), Front (F), Front (F), Back (B)”. This method was used because the leveling instrument checks whether the deviations between the BF and FB readings or between the FF and BB readings are not larger than expected. If this is the case, the leveling instrument warns the user and asks whether to ignore the deviation or remeasure that station. Therefore, a plausibility check during the measurements can be conducted. The instrument was stationed equidistant between two rods; this reduces the effects of refraction and Earth curvature.

During the post-processing, an adjustment was made. The loop error was distributed to the single measurements, inversely weighted by the distance between the instrument and the pole. To obtain the orthometric height, the height of the benchmark was taken and added to the height difference. The orthometric heights of the leveling markers are primarily from swisstopo’s latest line measurement across the Julier Pass in 1981 (with some later fixes where the points were destroyed). Because the distances between the auxiliary benchmarks and the stations were very short, it was deemed sufficient to use the orthometric height of the station and the leveled height difference without applying any corrections. The SD of the height difference (${SD}_{Niv,\;1km}$) was calculated as follows:

(4) $${SD}_{Niv,\;\;1km}=\sqrt{\frac{1}{4N}\sum_{j=1}^{N}\frac{{w_{j}}^{2}}{L_{j,km}}}$$

where N is the number of loops, $w_{j}$ is the loop error, and $L_{j,km}$ is the length of the leveling. The SDs of the benchmark heights were added to the leveled SDs to get integrated SDs for the auxiliary benchmarks. After the leveling data adjustment, the accuracy of the leveled mark is below 5 mm, and the largest SD of the height difference is about 1.1 mm.

### 2.4. Gravimeter Measurement

The *Scintrex CG-6* portable relative gravimeter was used for the gravimeter measurements. The operating principle of a relative gravimeter is that it measures a counterforce that must be applied to keep a test mass in an equilibrium position. When there are spatial or temporal changes in the gravity acceleration, the counterforce changes. The force to be applied can be converted to a gravity unit and calibrated with colocated absolute gravimeter measurements, which are direct measurements of the acceleration of gravity. Absolute gravimeter measurement is cumbersome and time-consuming, while relative gravimeter measurements can be obtained at high precision within minutes using a single operator. Various counterforces are used by different gravimeters. In the case of the CG-6 gravimeter, an electrostatic counterforce is applied [32].

The gravimeter measurements were intended to be conducted at all stations where GNSS and astrogeodetic measurements were obtained. Therefore, the absolute gravity must be known at the end of each observation station; since the relative gravimeter was used, we needed a connection station from which the absolute gravity is known. In the re-gion of the campaign, these are the first-order height control points (HFP1) where absolute gravity is known: in Tiefencastel, Savognin, Mulegns, Bivio, Silvaplana and on the Maloja pass. By including one of these stations, the gravity of the other stations can then be de-termined, since the gravity is measured relative to the connecting stations.

Eight sets of 30-s measurements for each station were conducted with an average of the 30-s measurements for each set written to a file. These sets of measurements were chosen because some of the stations were located along frequently traveled roads, which resulted in distorted measurements due to vibrations from passing vehicles, and thus the redundancy must be large enough to enable the deletion of certain sets in post-processing. Although there were eight measurements at each station, the last four of them at each station were very similar. Therefore, we used the last four gravity measurements at each station, which were then aggregated into a single mean for each station using their individual SDs as weights. To get the absolute gravity values from the relative gravity values $\left(g_{rel}\right)$, the instrument height must be corrected. It was corrected with 0.308 mGal/m so that the correction increased if the instrument was positioned higher. The instrument height ($H_{i}$) was measured to the bottom of the instrument and had to be corrected with 10.2 cm to get the height of the gravimeter. 

With the corrected values, the drift of the instrument could be determined. One method of drift (d) calculation utilizes the first and last measurements done on the same benchmark. The correction of the drift was added time depending on each measuring benchmark as follows:

(5) $$g_{rel,corr}=g_{rel}+0.308\;\frac{mgal}{m}\;\left(H_{i}-0.102\right)+d\;\frac{t_{meas}}{t_{tot}}$$

where $t_{meas}$ is the timestamp of the measurement and $t_{tot}$ is the duration between the measurements. Equation (5) demonstrates all the corrections for the relative gravity measurements with the resulting corrected relative gravity value $g_{rel,corr}$.

In this study, instead of using Equation (5), the gravity values $\left(g_{rel,corr}\right)$ were calculated using individual values interpolated for each station from a digital terrain model, which varied between 0.14 mGal/m in Tinizong and 0.36 mGal/m on the Maloja Pass. To achieve this, we used swissAlti3D (swissALTI3D from admin.ch) with a resolution of 2 m to reduce and interpolate the gravity values. Additionally, we used a bathymetry model for the lakes, mainly SwissBathy3D (swissBATHY3D from admin.ch). We also had models for local artificial reservoirs (such as Lake Marmorera), which were obtained directly from the hydroelectric companies. More specifically, we calculated complete Bouguer anomalies (using an assumed standard density of 2.67 g/cm^3^) at the points where we wanted to interpolate, then interpolated the Bouguer anomalies (using least squares collocation) at new points, and finally calculated absolute gravity values at these points. Since several benchmarks with known absolute gravity were included in the profile, an adjustment was performed to obtain the constant offset between relative and absolute gravity ($c_{rel,abs})$. With this known constant, it is possible to determine the absolute gravity ($g_{abs})$ of all relative measurements as follows:

(6) $$g_{abs}=g_{rel,corr}+c_{rel,abs}$$

We achieved an interpolation accuracy of approximately 1 mGal in difficult terrain. This accuracy is usually sufficient for reducing leveling data and calculating geopotential numbers.

## 3. Methodology

**Figure 2:**
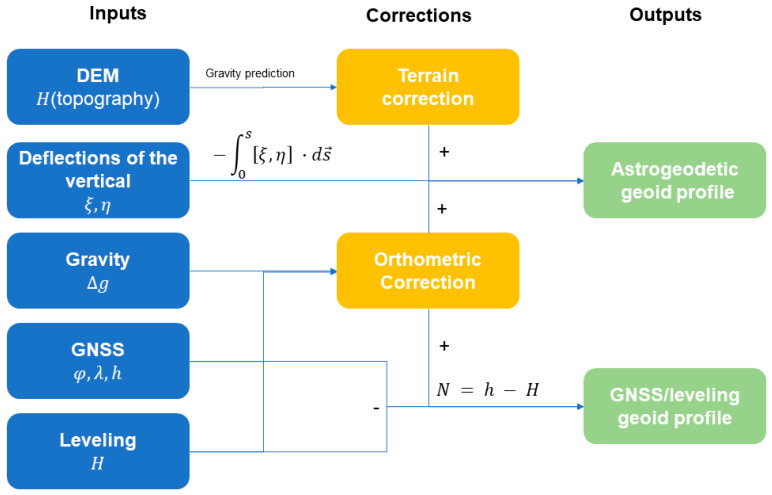
The data collection and analysis scheme for this study, including the types of datasets used and the flow of information in the reduction and processing of these data points into geoid profiles.

Two different conventional methodologies were carried out to determine geoid heights using collected data in the Surses region: GNSS/leveling and astronomical leveling (from DoV) methods. It should be noted that, by using the astronomical leveling method, we calculate the geoid height differences. To distinguish between these two methods, we referred to the direct geoid height determinations as “absolute” and the geoid height differences as “differential”. The data collection, reduction, and analysis scheme is illustrated in the block diagram in Figure 2. In addition to these two methods, CHGeo2004, EGM2008, and XGM2019e-predicted geoid heights were also used for comparison. All geoid height calculation methods used in this research are explained in this section.

### 3.1. Geoid Height Determination by GNSS/Leveling

The geoid height (N) from GNSS (ellipsoidal height h) and leveling (orthometric height H) data can be obtained as follows:

(7) N=h−H

In Equation (7), the orthometric heights can be determined in different ways. We used the following two approaches to obtain the orthometric heights of the auxiliary marks:

(1) The orthometric heights of the HFP1 marks ($H_{ort,HFP1}$) were obtained from the *Swiss Federal Office of Topography swisstopo*, which contained all possible corrections. To obtain the orthometric heights of the auxiliary marks ($H_{orth}$), the leveled height differences (${\Delta H}_{lev}$) were added to the orthometric heights of the marks ($H_{orth,HFP1}$):
  (8) $$H_{orth}=H_{orth,HFP1}+{\Delta H}_{lev}$$
(2) The orthometric heights can also be obtained from the *REFRAME* online service from the *swisstopo* website (swisstopo.admin.ch). The GNSS coordinates were used and transformed to the reference frame Land Survey 1995 (LV95). Then, leveled heights ($H_{lev}$) were inserted, since they are more accurate. Finally, the heights could be transformed in the Swiss National Height Network (LHN95), resulting in LHN95 orthometric heights ($H_{orth,CHGeo2004}$).

As a result of these two orthometric height calculations, two different geoid heights were obtained: $N_{GNSS/leveling}$ from $H_{orth}$ and $N_{CHGeo2004}$ from $H_{orth,CHGeo2004}$.

### 3.2. Geoid Height Determination by Astronomical Leveling

The geoid height differences (ΔN) can be calculated from astrogeodetic DoV data using astronomical leveling, as follows:

(9) $$\Delta N_{AB}=N_{B}-N_{A}=-\int_{A}^{B}\varepsilon ds$$

ε: projected astrogeodetic DoV along the integration path

ds: the length of the path increment between (adjacent) stations A and B

ΔN: the geoid height differences

In astronomical leveling, it is necessary to obtain the GNSS, leveling, and gravity data at each of the DoV observation locations. These measurements can be conducted either before or after the DoV observations (see Section 2). As a conceptual starting point, the geoid height from the astrogeodetic technique can also be determined differentially between two points, *i* and *k*. Since the distance between the two adjacent stations is small, the differential geoid height can be calculated as follows [33]:

(10) $$\Delta N_{ij}=N_{j}-N_{i}=-\left(\frac{\xi_{i}+\xi_{j}}{2}\;\cos\alpha_{ij}+\frac{\eta_{i}+\eta_{j}}{2}\;\sin\alpha_{ij}\right)s_{ij}$$

where ξ and η are the respective N-S and E-W DoV components of the stations (see Equations (1) and (2)) between which the differential geoid height is to be determined. The information about the azimuth ($\alpha_{ij}$) as well as the slope distance ($s_{ij})$ between the two points *i* and *j* are calculated using geodetic coordinates from the GNSS measurements.

Equation (10) applies to DoV measurements taken at or near the geoid. However, because of the wide variation in orthometric height and the average height being in excess of 1 km in this study, the DoVs we measured are more reflective of the normal vectors of a disparate set of equipotential surfaces that are not parallel to the geoid.

It is more rigorous to write Equation (9) in terms of the disturbing potential, defined by Bruns’ equation and the fundamental equation of physical geodesy, as follows:

(11) $$\Delta T_{AB}=T_{B}-T_{A}=-\int_{A}^{B}\gamma \varepsilon ds-\int_{A}^{B}\delta gdh$$

δg is the gravity disturbance, or the observed gravity minus the normal gravity acceleration γ according to the latitude and orthometric height of the station. The gravity disturbance is the negative of the vertical component of disturbing potential’s gradient in a similar way to how −γε gives the projected horizontal component. In the final term of Equation (11), the gravity disturbance is integrated across increments of ellipsoidal height to give the *dynamic correction*. In our study, the dynamic correction is small, with an SD of 6 mm.

The disturbing potential is valid for the point at the surface where the DoV and gravity measurements were made. Dividing the disturbing potential by γ at the location of the measurement gives the quasigeoid ζ. The quasigeoid is not an equipotential surface and it differs from the geoid by decimeters in mountainous terrain due to the potential introduced by the mass between the geoid and the topographic surface. We therefore apply the geoid-quasigeoid separation correction to our geoid profile [34]. The effect of this term introduces a slope of approximately 6 cm between the first and last benchmarks of our profile.

(12) $$N-\zeta =\frac{\bar{g}-\bar{\gamma }}{\bar{\gamma }}H\approx \frac{\delta g_{B}}{\gamma }H$$

In this scheme, $\bar{g\;}$ and $\bar{\gamma }$ are the mean total and normal gravity between the observation point and the geoid. It is more practical to substitute and $\bar{g}-\bar{\gamma }$ with the Bouguer disturbance $\delta g_{B}$ using the observed gravity, accounting for the Bouguer plate [35] and the normal gravity at the observation point γ. The complete geoid increment observation equation is therefore modified to the following:

(13) $$\Delta N_{AB}=N_{B}-N_{A}=-\int_{A}^{B}\varepsilon ds-\int_{A}^{B}\frac{1}{\gamma }\Delta gdh+\frac{\delta g_{B,B}}{\gamma_{B}}H_{B}-\frac{\delta g_{B,A}}{\gamma_{A}}H_{A}$$

Note that in this equation, dividing by the height-dependent term γ replaces the gravity disturbance δg with the familiar gravity anomaly Δg. In practice, this equation is discretized to match the trapezoid integration approach of Equation (10). In our computation scheme, the s term representing the distance between the observations is computed from the meridional and prime-vertical radii of curvature of the reference ellipsoid at the mean altitude of the segment. Before populating the equation with the appropriate ε values, the DoV data require corrections for the curvature of the plumbline. The dynamic correction and geoid-quasigeoid separation correction account for the corrections for the curvature of the plumbline in the disturbing field in integral form, but do not account for the ellipsoidal contribution. Following Hofmann-Wellenhof and Moritz [36], an ellipsoidal plumbline curvature correction of −0.17″/km Hsin⁡2φ was added to the ξ measurements. These plumbline-corrected measurements were run through Equation (13) to obtain the initial geoid increments.

### 3.3. Geoid Height Determination Using CHGeo2004, EGM2008 and XGM2019e

In this research, we used predicted geoid heights from the latest Swiss national geoid model CHGeo2004 [12,37], and two global gravity field models, EGM2008 [25] and XGM2019e [26], for comparison against GNSS/leveling- and DoV-derived geoid heights. CHGeo2004 can be accessed from the swisstopo website (https://www.swisstopo.admin.ch/en/geoid-en: 18 September 2024), while EGM2008 and XGM2019e are available from the International Centre for Global Earth Models (ICGEM, http://icgem.gfzpotsdam.de/: 2 September 2024) website [38]. Both EGM2008 and XGM2014e are entirely independent of our astrogeodetic DoV datasets. The geoid heights from these models were computed at the benchmark locations using the geopotential functional calculation service available on the ICGEM website.

CHGeo2004 was determined by combining many different datasets, comprising 30,000 gravity, 700 DoV, and 200 GNSS/leveling data points for the entirety of Switzerland [12,37]. It also includes 270 GNSS/leveling data points from neighboring countries. In addition to combining these data to create the geoid, Marti [12] also individually calculated the astrogeodetic, gravimetric, and GPS/leveling solutions. The official CHGeo2004 model, which is used mainly by surveyors to transform the GPS heights to orthometric heights, incorporate GPS/leveling data with high weights applied. The main differences between the CHGeo2004 model and other national geoid models are that the CHGeo2004 includes the DoV data and the reduction of observations was conducted using a simple 3D density model of the Earth’s crust with regular reductions from global geopotential model (EGM96) and a digital terrain model [12,37]. Although the calculation of the model is based on the least squares collocation, the parameters of the covariance function have been modified slightly to minimize the resulting residuals between the astrogeodetic, gravimetric, and the GPS/leveling geoid models. As a result, the CHGeo2004 accuracy was determined as 2–3 cm in the most parts of Switzerland, but the accuracy of the model is decreased in the mountainous regions, where it may vary between 5–10 cm.

The EGM2008 model was developed using gravity anomalies from terrestrial observations, satellite-to-satellite tracking, and multi-mission radar altimetry over ocean areas [20], and has been widely used as a reference model in geodetic and geophysical applications as diverse as potential field modeling, gravity interpretation, and gravity inversion (e.g., [39,40,41,42,43,44,45,46]). Many astrogeodetic research projects have incorporated accuracy estimations of the EGM2008 model, for instance, [11,47].

XGM2019e is one of the recent combined global gravity field models [26]. It was developed from spheroidal harmonics up to degree and order 5399, corresponding to a spatial resolution of 2′ (~4 km). It includes ground and ocean data from various sources: satellite model GOCO06s with a ground gravity grid, NGA’s land and ocean of gravity anomalies with topographically derived gravity information over land (EARTH2014), and DTU13 gravity anomalies derived from satellite altimetry over the ocean. All calculations were performed in the spheroidal harmonic domain.

## 4. Results

The DoV, GNSS, leveling, and gravimeter data collection methods and how their SDs were individually calculated were explained in Section 2. As seen in Section 3, we used GNSS, leveling, and DoV data for independent geoid height calculations. The relative gravimeter measurements in the Surses profile were collected along a single one-dimensional profile, precluding Stokes integration, and were not sufficient to generate a geoid profile on their own without external statistical assumptions (e.g., least-squares collocation). Instead, the relative gravity data were used to inform elevation-dependent corrections to the astrogeodetic geoid profile, such as the geoid-quasigeoid separation term.

### 4.1. Gravimeter Measurements Results

**Figure 3:**
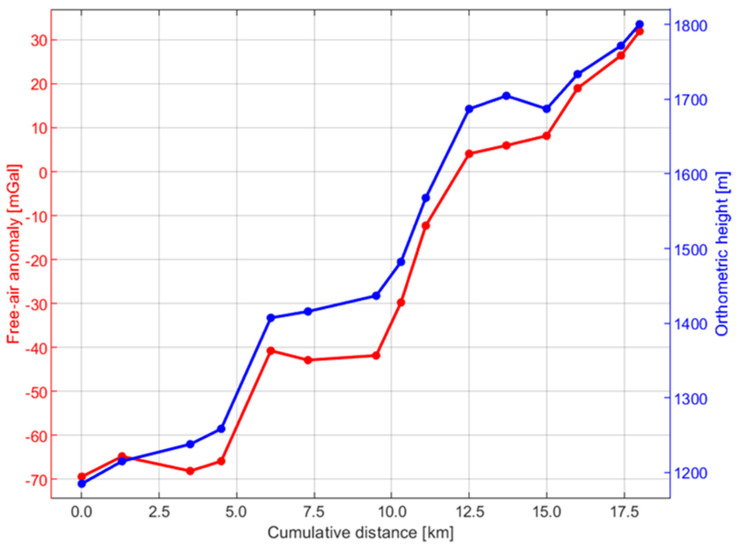
Free-air anomaly (red) versus the orthometric heights (blue).

The gravimetric data were primarily used for DoV and orthometric height corrections. To calculate a gravimetric geoid, collected gravimeter data should be distributed across two dimensions rather than a profile, and furthermore, the terrain model must be taken into account. We calculate both free-air anomalies and disturbances from the gravity data using a rigorous realization of the GRS80 normal gravity formula. The gravimetric data are sensitive to changes in height (in this case, orthometric height), as can be seen in Figure 3.

Relative gravimeter measurements at each benchmark were transformed into full-field gravity data (see Section 2.4). These gravity observations were used for corrections to the geoid profile. The gravimeter and DoV measurements are affected by the changes in the water level in the nearby Marmorera Lake. Therefore, impacts of the reservoir water level were calculated, and the appropriate corrections for gravimeter and DoV data were made (Appendix A). Per the inverse square law, these corrections had the highest impact on benchmarks closest to the lake. The highest gravimeter and DoV corrections were conducted for benchmark number 10, which is near the dam. The reason why this mark is most affected could be that the lake is deepest at the dam.

**Figure 4:**
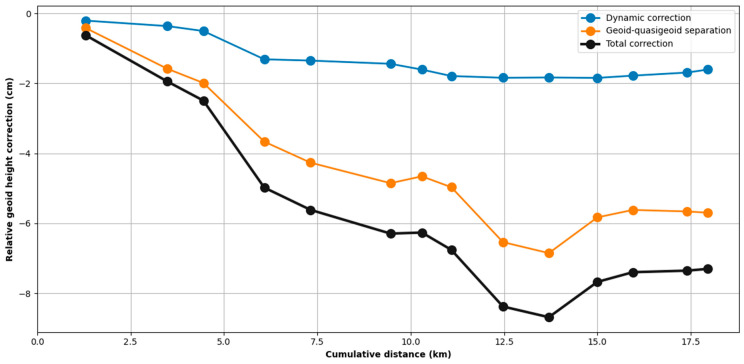
Geoid profile corrections (relative to the first mark) added to the quasigeoid profile derived from observed deflections of the vertical (DoV). These include the dynamic correction derived from observed gravity disturbances and the geoid-quasigeoid separation term.

The effect of the dynamic correction and geoid-quasigeoid separation are illustrated in Figure 4, which shows the relative geoid height corrections as a function of cumulative distance along the 18 km profile. The dynamic correction (blue line) remains relatively constant across the profile, indicating that the dynamic effects on the geoid height are stable throughout the measurement path. In contrast, the geoid-quasigeoid separation (orange line) exhibits a more variable trend that mimics the topography, introducing a correction of nearly −6 cm between the first and last marks of the profile. The sum of these corrections, the orthometric correction (black line), changes the quasigeoid profile by up to −8 cm. This figure illustrates the importance of applying both dynamic and geoid-quasigeoid separation corrections to astrogeodetic results to achieve accurate geoid height determinations, particularly along profiles where elevation changes by hundreds or thousands of meters.

### 4.2. Absolute Geoid Height Results

**Figure 5:**
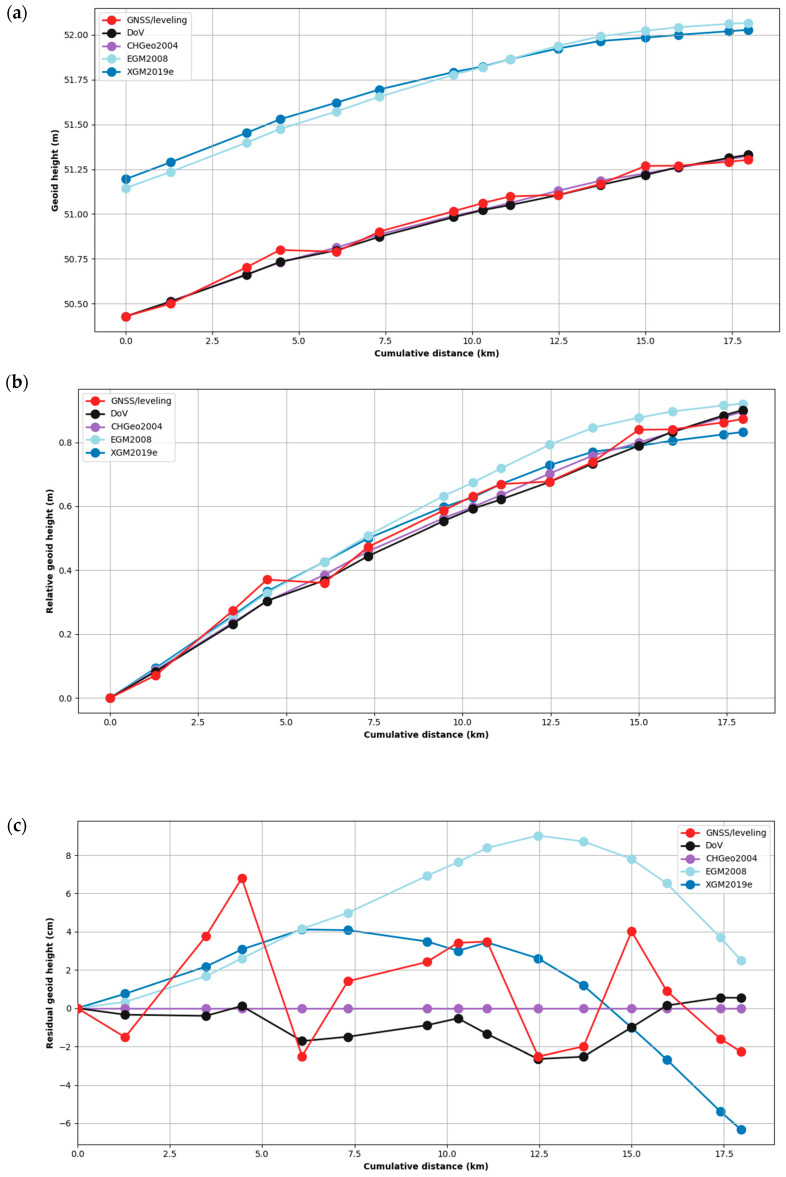
(**a**) Absolute geoid height results from GNSS/leveling and DoV compared with CHGeo2004, EGM2008, and XGM2019e. (**b**) The same results relative to the first mark in the profile. (**c**) The same results minus CHGeo2004.

Two different GNSS and leveling-derived geoid heights were calculated: $N_{GNSS/leveling}$ and $N_{CHGeo2004}$ (see Section 3.1). $N_{GNSS/leveling}$ was derived using our GNSS and leveling measurements, while $N_{CHGeo2004}$ was based on the CHGeo2004 model. The accuracy of the CHGeo2004 is on the order of 2–3 cm in most parts of Switzerland. Therefore, the geoid model can be validated with the independent calculation of N, since the deviations of $N_{GNSS/leveling}$ and $N_{CHGeo2004}$ are in the range of the accuracy of the geoid model. The results for calculated geoid heights from GNSS and leveling, CHGeo2004, DoV, EGM2008, and XGM2019e can be seen in Figure 5. The SDs of the differences between all absolute geoid profiles in this study are shown in Table 1.

### 4.3. Geoid Height Increments

**Figure 6:**
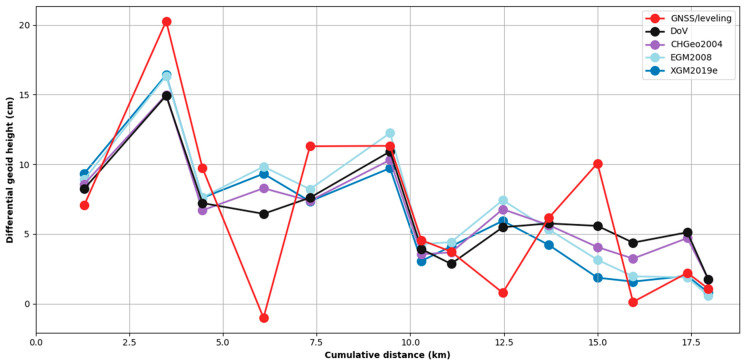
Differential geoid height calculation results from DoV, GNSS/leveling, EGM2008, and XGM2019e. To remove long-wavelength signals, each point shows the difference in geoid height between a point and the previous point in the survey.

Geoid heights computed from DoV are based on mark-to-mark differential increments integrated along the profile (see Section 3.2). Integration tends to suppress high-frequency features in the data and makes it difficult to assess the dataset visually. Therefore, to compare the DoV-derived geoid height results with other results (GNSS/leveling, CHGeo2004, EGM2008, and XGM2019e), we computed the mark-to-mark geoid increments to match the corrected astrogeodetic data. These results can be seen in Figure 6. Each point in Figure 6 represents the mark-to-mark differential geoid undulation as indicated by the average DoV measured at this point and its predecessor in the survey sequence according to Equation (13). This differential approach allows us to localize measurement errors and suppress the long-wavelength trend that dominates geoid undulation along the profile.

## 5. Discussion

Our work has generated two novel geoid profiles in this region using GNSS/leveling and astronomical leveling augmented by gravity observations. These profiles are presented in both absolute terms and differential (mark-to-mark) increments. These results were compared with CHGeo2004, EGM2008, and XGM2019e. The absolute geoid heights show a general upward trend along the cumulative distance (Figure 5a), indicating that the geoid surface rises progressively along the profile. Figure 5b shows the same results relative to the first mark to show the relative agreement of these profiles.

The absolute geoid heights calculations using GNSS/leveling data were based on GNSS data. As the accuracy of the GNSS data was at the centimeter level and the relative leveling precision across the profile is several millimeters, ellipsoidal height accuracy seems to the be limiting factor in the GNSS/leveling profile. The geoid height accuracy of the GNSS/leveling is therefore expected to be in the centimeter range. Figure 5 shows that the CHGeo2004 predicts the GNSS/leveling results with a SD of 3 cm and aligns comparably well with the geoid heights derived from DoV measurements (also 3 cm). The harmony between these three profiles is unsurprising given that CHGeo2004 includes both DoV and GNSS/leveling data in Switzerland. However, the predicted geoid heights of both global gravity field models (EGM2008 and XGM2019e) differ significantly from the GNSS/leveling, with SDs closer to 4 cm. This is primarily explained by the limited spatial resolution of the global models, which is comparable to the length of the profile.

Local, short-wavelength variations are easier to see by comparing geoid height increments in Figure 6. It is immediately clear in this plot that the GNSS/leveling profile is the least consistent with the preponderant character of the profiles considered in this work. This is in part because the differencing operation adds the noise of two ellipsoidal height measurements. Both measured profiles show similar levels of disagreement with EGM2008 and XGM2019e also show better agreement with the astronomical data than the GNSS/leveling. In Figure 6, it is clear that the GNSS/leveling differential geoid height does not agree well with other models. While the leveling component of GNSS/leveling has sub-millimeter precision across these kilometer-scale baselines, the limiting factor is the ellipsoidal heights, which have greater than 1 cm noise. The differential view has the advantage of averaging the astronomical measurements across points, while differencing GNSS measurements tends to add noisy ellipsoidal heights together. These results highlight one advantage of using astrogeodetic methods for recovering geoid undulation over short baselines compared with GNSS/leveling methods.

We attribute the short-wavelength errors in the GNSS/leveling to noise in the GNSS ellipsoidal heights. However, errors in the reductions and adjustment of LHN95 may also contribute to this profile. While we spent some time investigating whether our new gravity data could improve the orthometric heights, we ultimately chose to keep the profile as-is to reflect the height accuracy of the LHN95 network.

This study may be compared with the more extensive GSVSs of 2011 [8], 2014 [9], and 2017 [10]. These surveys used DIADEM and later CODIAC astrogeodetic observations in concert with GNSS, gravity, and leveling to generate geoid profiles of approximately 300 km in length with a standard spacing of approximately 1.6 km. The GNSS/leveling in these studies was conducted on a larger scale than ours with original marks set, longer GNSS occupations (40+ h), and original leveling across the entire profile, rather than ties to an existing network with historical leveling.

The most challenging of these surveys was GSVS17 in the mountains of Colorado, where extreme topographic variation required attentive processing of refraction errors in leveling. The independent DoV and GNSS/leveling profiles in GSVS17 showed RMS agreement better than 2 cm across the entirety of the 360 km survey, indicating the accuracy of both techniques as a means of profiling the geoid and recovering geopotential differences.

Our study is conducted with a much more limited spatial scope of 18 km, but a higher density of marks. Comparing our results to these illustrates the tradeoffs of using shorter GNSS occupations and historical leveling networks to efficiently gather data for geoid validation. The time and labor demands of high-quality GNSS/leveling for geoid validation highlights the logistical and observational robustness of geodetic astronomy as an alternative.

## 6. Conclusions

In this research, our goal was to provide a geoid determination solution to the mountainous Surses region in the Eastern Swiss Alps, where problems in regional gravity field determinations and height unification systems have long existed. This project amassed measurements using astrogeodetic systems, GNSS, leveling, and a gravimeter on a North-South profile along the Julier Valley, which are significant to determine a new geoid in this region where existing datasets cannot provide sufficient accuracy. DoV data are particularly important in mountainous areas, are the most important data for comparative analysis, and can enable the validation of geoid models and global gravity field models from other datasets. DoV data can also be used to indicate possible shortcomings in official geoid and regional gravity field models and height systems, and to validate those models.

Geoid determination in the Surses region was carried out by obtaining independent GNSS/leveling and DoV data. These geoid heights were compared with CHGeo2004, EGM2008, and XGM2019e. According to the resulting comparisons of the absolute and differential GNSS/leveling geoid heights, the absolute GNSS/leveling geoid heights capture the long-wavelength character of CHGeo2004 model (Figure 5), but the differential GNSS/leveling geoid height increments are noticeably noisier compared with the other profiles (Figure 6), highlighting the impact of the GNSS accuracy on GNSS/leveling observations. This short-wavelength noisiness limits the utility of GNSS/leveling for gravity modeling at kilometer scales. This limitation matters less at longer scales (>10 km), where the integration error of both DoV and leveling approach uncertainties in ellipsoidal heights. At regional and continental scales, the dominant error source in GNSS/leveling is systematic errors in leveling rather than GNSS.

The astrogeodetic measurements were more accurate for defining the differential geoid height than the absolute geoid height. The third component of this study was the CHGeo2004 model verification for the study area at a centimeter level accuracy. Our campaign confirmed the accuracy at the centimeter level for this model. We also showed that the global gravity field models EGM2008 and XGM2019e do not agree well with the measurements for the absolute geoid height determinations, and they gave better agreement when differential geoid heights were used.

DoV data have not been previously collected in the Surses region, and the 700 DoV data points used to determine the CHGeo2004 were excluded in this mountainous region. While CHGeo2004 has high accuracy (2–3 cm) in most parts of Switzerland, the accuracy of the model in the mountainous region is between 5 and 10 cm. Therefore, our new geoid determination in this region may also contribute to subsequent versions of the CHGeo2004. Additionally, the methods developed in this study are currently being extended to a project validating the GEOID2022 model developed by NOAA’s National Geodetic Survey as part of the modernization of the National Spatial Reference System (NSRS).

## Figures and Tables

**Table 1:** Standard deviations (SDs) of the differences between all geoid profiles in this study (centimeters).

	DoV	GNSS/Leveling	CHGeo2004	EGM2008	XGM2019e
GNSS/leveling	3.0	0			
CHGeo2004	1.0	3.0	0		
EGM2008	3.6	4.1	3.1	0	
XGM2019e	4.2	3.6	3.3	4.0	0

## Data Availability

The original contributions presented in the study are included in the article; further inquiries can be directed to the corresponding author.

## Appendix A

**Table A1:** Measured geodetic coordinates (in ITRF2014) and North-South (ξ) and East-West (η) components of astrogeodetic deflections of the vertical (DoV), along with the corresponding standard deviations (SDs).

BM	Geodetic Coordinates		Mean DoV Values		SD for DoV	
	φ [°]	λ [°]	ξ [″]	η [″]	ξ [″]	η [″]
1	46.608297	9.591780	11.86	−9.18	0.05	0.01
2	46.597509	9.597783	12.09	−8.86	0.18	0.20
3	46.583273	9.617558	9.57	−13.18	0.15	0.16
4	46.576381	9.625470	11.76	−13.21	0.02	0.02
5	46.561838	9.623816	13.16	−8.28	0.12	0.12
6	46.550780	9.624950	13.73	−9.02	0.04	0.11
7	46.531568	9.622235	10.29	−2.77	0.18	0.09
8	46.524085	9.620596	10.08	−1.63	0.09	0.07
9	46.520307	9.629100	9.54	−7.22	0.14	0.03
10	46.508839	9.635777	7.02	−9.74	0.22	0.09
11	46.498598	9.641930	7.08	−10.37	0.05	0.07
12	46.486885	9.641337	8.54	−4.97	0.02	0.02
13	46.478743	9.645076	7.46	−5.42	0.13	0.06
14	46.467116	9.653650	3.52	−5.98	0.19	0.12
15	46.463371	9.658366	3.51	−5.87	0.08	0.19

**Table A2:** Corrected deflections of the vertical (DoVs) and gravity considering the impact of the water height of the reservoir.

	Impact of Reservoir Water Height				Corrected DoV		Corrected Gravity
BM	Water Height	ξ	η	Gravity	ξ_corr._	η_corr._	Gravity_corr._
	[m]	[″]	[″]	[mGal]	[″]	[″]	[mGal]
1	1665.75	0	0	−0.0001	11.86	−9.18	980,330.3190
2	1665.75	0	0	−0.0001	12.09	−8.86	980,324.6941
3	1659.08	0	0	−0.0001	9.57	−13.18	980,313.0320
4	1665.75	0	0	−0.0002	11.76	−13.21	980,308.2795
5	1659.08	0	0	−0.0002	13.16	−8.28	980,286.2851
6	1661.56	0	0	−0.0004	13.73	−9.02	980,280.5180
7	1658.71	0	0	−0.0014	10.29	−2.77	980,273.3360
8	1659.08	0.01	0	−0.0024	10.09	−1.63	980,270.7577
9	1661.56	0.01	0	−0.0027	9.55	−7.22	980,261.4670
10	1659.08	0.16	0.06	0.2382	7.18	−9.68	980,240.0806
11	1661.56	−0.01	0.12	0.1329	7.07	−10.25	980,235.6146
12	1665.75	−0.03	0.01	0.0085	8.51	−4.96	980,242.1410
13	1661.56	−0.01	0	0.0017	7.45	−5.42	980,237.9077
14	1659.08	0	0	0.0006	3.52	−5.98	980,232.5479
15	1661.56	0	0	0.0005	3.51	−5.87	980,228.8645
